# Breath-hold and free-breathing F-18-FDG-PET/CT in malignant melanoma—detection of additional tumoral foci and effects on quantitative parameters

**DOI:** 10.1097/MD.0000000000005882

**Published:** 2017-01-13

**Authors:** Robert Bärwolf, Mariana Zirnsak, Martin Freesmeyer

**Affiliations:** Clinic of Nuclear Medicine, Jena University Hospital, Jena, Germany.

**Keywords:** additional finding, breath-hold PET/CT, F-18-FDG-PET/CT, metastasized malignant melanoma, quantitative parameter

## Abstract

During PET/CT acquisition, respiratory motion generates artifacts in the form of breath-related blurring, which may impair lesion detectability and diagnostic accuracy. This observational study was undertaken to verify whether breath-hold F-18-FDG-PET/CT (bhPET) detects additional foci compared to free-breathing PET/CT (fbPET) in cases of malignant melanoma, and to assess the impact of breath-holding on standard uptake values (SUV) and metabolic isocontoured volume (mV_ic40_).

Thirty-four patients with melanoma were examined. BhPET and fbPET findings of 117 lesions were compared and correlated with standard contrast-enhanced (ce) CT and MRI for lesion verification. Quantitative parameters (SUV_max_, SUV_mean_, and mV_ic40_) were assessed for both methods and evaluated by linear regression and Spearman correlation. The impact of lesion size and time interval between investigations was analyzed.

In 1 patient, a CT-confirmed liver metastasis was seen only on bhPET but not on fbPET. At bhPET, SUV_max_, and SUV_mean_ proved significantly higher and mV_ic40_ significantly lower than at fbPET. The positive effect on SUV_max_ and SUV_mean_ was more pronounced in smaller lesions, whereas the time interval between bhPET and fbPET did not influence SUV or mV_ic40_.

In our patient cohort, bhPET yielded significantly higher SUV and provided improved volumetric lesion definition, particularly of smaller lesions. Also one additional liver lesion was identified. Breath-hold PET/CT is technically feasible, and may become clinically useful when fine quantitative evaluations are needed.

## 1 Introduction

The F-18-fluorodeoxyglucose positron emission tomography (F-18-FDG-PET) is an established method for the imaging of malignant melanoma.^[1,2]^ In contrast to computed tomography (CT) and magnetic resonance imaging (MRI), PET scanning cannot be routinely performed in breath-hold mode because of the required duration of several minutes. However, performing the scans in free-breathing generates artifacts in the form of breath-related blurring.^[3]^

Two methods have been tested to limit the impact of breath-related blurring in the application of F-18-FDG-PET/CT: respiratory gating (rgPET) and breath-hold PET (bhPET).^[4,5]^

Disadvantages of rgPET are the need of dedicated hardware and software, the technical effort and costs, the long scanning time, a high radiation exposure, and prolonged reconstruction times. These problems have resulted in limited application of rgPET in clinical routine.^[6,7]^ In contrast, bhPET has no additional costs and requires only limited effort, but image quality largely depends on the ability of the patient to hold his breath.^[8]^ For chest findings, in particular, investigations on the effects of bhPET/CT with F-18-FDG have shown that the standard uptake value (SUV) is typically higher and the isocontour volume smaller than with free-breathing (fb) PET/CT.^[9,10]^ The ability to detect additional tumoral foci, normally missed by the free-breathing technique, has been demonstrated in a case of carcinomatous lymphangitis^[8]^ and in a liver metastasis of a colorectal carcinoma.^[11]^

The goals of this study were to evaluate whether F-18-FDG bhPET/CT allows the detection of additional tumoral foci in patients with melanoma compared with fbPET/CT, and to verify the impact of breath-holding and free-breathing on quantitative parameters.

## 2 Materials and methods

### 2.1 Patients, lesions, and ethics

The observational study sample comprised 34 consecutive adults (23 males/11 females; mean age 60 years, range 22–82), with clinically and histologically confirmed metastatic melanoma (Table 1). Over a period of 3 years, the patients were referred to our center for F-18-FDG-PET/CT aimed at tumor staging, restaging, and surveillance. Including follow-up investigations, overall 46 studies were performed and 117 lesions were evaluated (Table 2). All data were analyzed retrospectively.

**Table 1:**
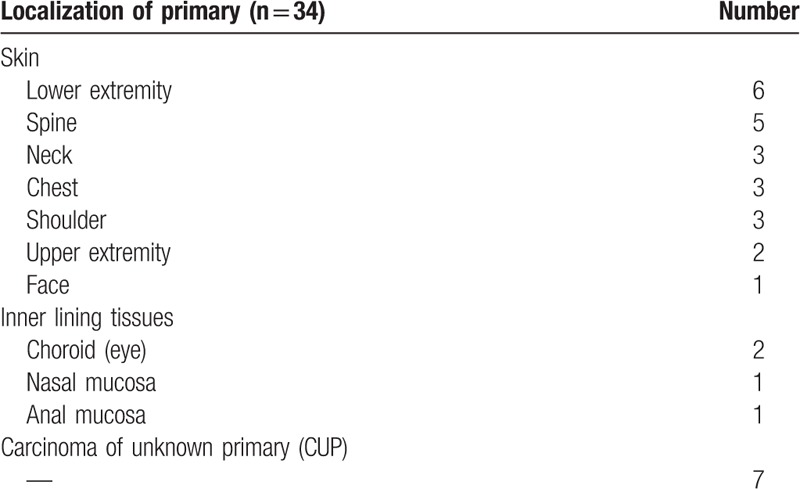
Localization of primary tumor.

**Table 2:**
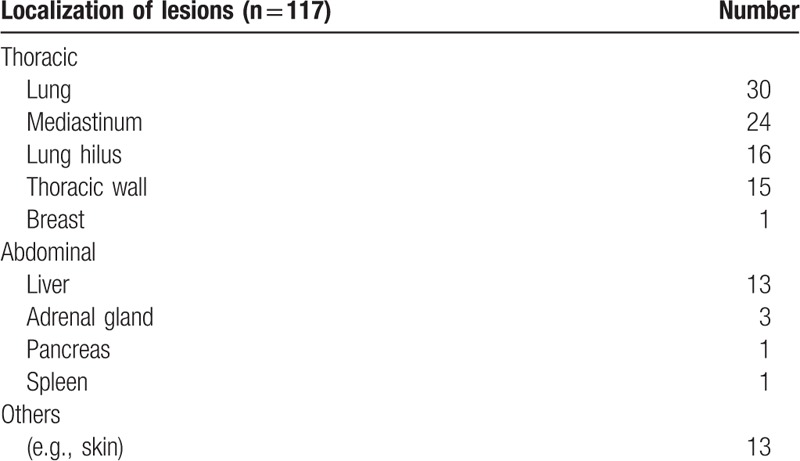
Localization of lesions detected by fbPET and bhPET (without lesion detected by bhPET only).

The study was approved by the local ethics committee, and all patients signed a written informed consent.

### 2.2 F-18-FDG administration

The preadministration blood glucose levels were 5.6 ± 1.2 mmol L^−1^ (mean ± SD). Diabetes mellitus type II was present in 5/34 (14.7%) patients (both tablet- and insulin dependent). 264 ± 18 MBq F-18-FDG (7.13 ± 0.49 mCi) was administered in 10 mL of 0.9% saline as a bolus, followed by flushing with the same amount and concentration of saline according to current guidelines.^[12]^ Scanning procedures were performed 101 ± 24 minutes (range 56–180 minutes) after tracer injection.

### 2.3 fbPET/CT

Each patient was positioned in the PET/CT system (Biograph mCT 40 with a TrueV fourth PET ring and a 21.8 cm axial field-of-view; Siemens, Erlangen, Germany) with arms beside the body and in supine position. Automatic voice announcements instructed the patient to breath in a regular and shallow fashion. A noncontrast low-dose CT scan for attenuation correction and anatomical reference was obtained (50 mAs, 120 kV tube voltage, 3 mm slice thickness, 2 mm increment). Whole-body fbPET scans were then performed, extending from the vertex to the feet. In total 12 to 14 bed positions were acquired (2 minutes from vertex to pelvis, 1 minutes for legs and feet, total 20–24 minutes).

### 2.4 bhPET/CT

A bhPET/CT including one bed position was performed 31 ± 7 minutes (range 17–60 minutes) after the fbPET/CT. In each case the localization of the breath-hold bed position was selected based on the clinical indication and query of the referring physician. In total 86 thoracic and 31 extrathoracic lesions were examined (Table 2).

First, a bhCT was performed in deep end-inspiration. CT parameters were the same as for the fbPET/CT scan. For the acquisition of bhPET, the patient was instructed to repeat the breathing exercise in the same way as long as possible. This phase was supervised by a radiographer and the scans were manually stopped when the patient resumed respiration.

### 2.5 Morphological imaging

Morphological sectional images used for method correlations were contrast-enhanced CT (ceCT) in 39/46 investigations (84.8%) and contrast-enhanced magnetic resonance imaging (ceMRI) in 1/46 investigations (2.2%). In 6/46 investigations (13%) ceCT could not be performed for the following reasons: renal insufficiency, allergy to contrast media, and lack of recent laboratory tests (TSH and/or glomerular filtration rate). For these patients only a low-dose CT (ldCT) was available.

A ceCT was performed in parallel to the PET/CT in 32/39 investigations (82.0%), whereas in 7/39 investigations (18.0%) recent ceCT examinations were considered for comparisons. The mean time interval between ceCT and PET was 10.9 ± 27.6 days. Follow-up examinations were available for 21/34 patients (61.8%).

### 2.6 Data analysis/quantitative parameters

Comparisons were performed by an experienced nuclear medicine specialist assisted by a doctoral candidate on a consensus basis. Data were presented on a dedicated multimodal evaluation console (Syngo MMWP Version VE31A, Siemens) with the aid of software for PET/CT analysis, and were visually assessed for increased tracer accumulation. First the attention was aimed at detecting focal findings seen at bhPET but not at fbPET. Then a targeted comparison was performed with the available CT and MRI in order to identify subtle morphological correlates. A 3-dimensional volume of interest (VOI) was drawn over the entire lesion of interest. Maximum and mean standardized uptake values (SUV_max_ and SUV_mean_) and metabolic isocontoured volumes (mV_ic40_) were assessed in both investigations.

The SUV was determined by dividing the measured tracer concentration by total injected activity and body weight. The SUV_max_ was derived from the single voxel with the highest tracer uptake within a VOI, thus avoiding a bias introduced by the VOI size with inclusion of a greater or smaller proportion of voxels of more intense or less intense uptake. The SUV_mean_ was derived from all voxels within the VOI, assuming that this more closely reflected the tracer uptake in that VOI, as seen with the human eye. The metabolic volume mV_ic40_ was defined as the metabolic isocontoured volume including all voxels exceeding 40% of the SUV_max_.

The percentage differences between bhPET and fbPET for SUV_max_, SUV_mean_, and mV_ic40_ were defined as follows: %bh-index = (bhPET − fbPET)/fbPET × 100.

### 2.7 Statistics

Differences between bhPET and fbPET measurements (SUV_max_, SUV_mean_, and mV_ic40_) were tested using 2-tailed and paired *t* tests. Associations were tested by linear regression and Spearman correlation (rho). The corresponding *P* values were additionally reported to avoid false correlations due to outliers. *P* values <0.05 were considered significant. Data were illustrated by Tukey box and whisker blots. Boxes represented the first, second, and third quartile, while the whiskers represented the lowest/highest value still within 1.5 interquartile ranges of the lower/upper quartile. Outliers were not shown.

All analyses were carried out using R, a free language and environment for statistical computing and graphics (R Core Team 2014).

## 3 Results

### 3.1 In comparison with bhPET/CT an additional lesion was detected that was not visible at fbPET/CT

**Figure 1:**
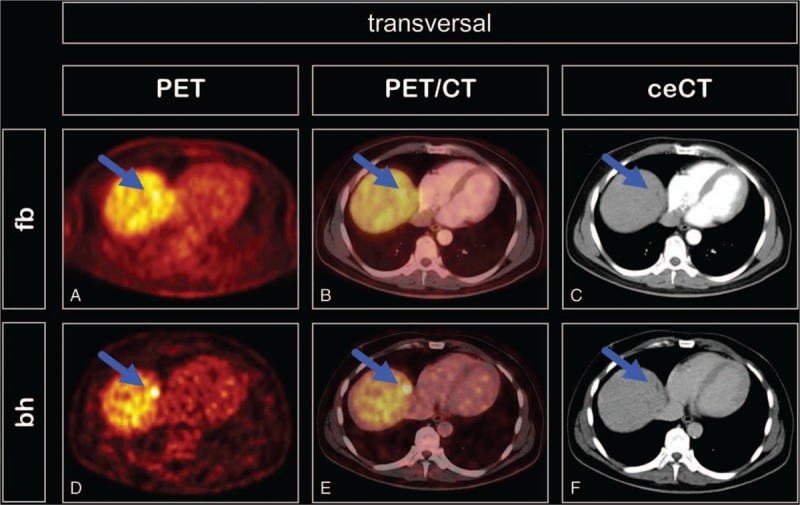
Transversal PET (A and D), PET/CT (B and E), and ceCT (C and F) images of a male patient with liver metastasis of malignant melanoma (liver segment II). The lesion (arrows) was not visible at free-breathing PET (A) but was evident at breath-hold PET (D). This lesion had a morphological correlate in the arterial (C) and late venous (F) phases of contrast-enhanced CT. Breath-hold time was 21 seconds, SUV_max_ was 13.3.

In the patient population analyzed in the present study, the bhPET identified 1 additional lesion not visible at fbPET but corresponding to a lesion identified at ceCT (Fig. 1). Because of the multifocal nature of the metastasized melanoma, a histological verification of the suspicious malignant lesion was not clinical appropriate, however a follow-up ceCT performed 2 months later confirmed the progression of the tumor. Overall, 117 and 118 lesions were detected both by fbPET and bhPET and by bhPET respectively, thus one lesion was only detected by bhPET.

### 3.2 Primary tumor and lesion localization of the patient cohort

The majority of primary tumors were located on the lower extremity (18%) and spine (15%). In 21% no primary could be identified (CUP) whereby diagnosis was confirmed by histological examination of metastases (Table 1). Malignant findings most frequently involved thoracic sites, for example, lungs (26%) and mediastinum (21%). In the abdomen, the liver was most commonly involved (11%, Table 2).

### 3.3 Impact of bhPET on quantitative parameters

**Figure 2:**
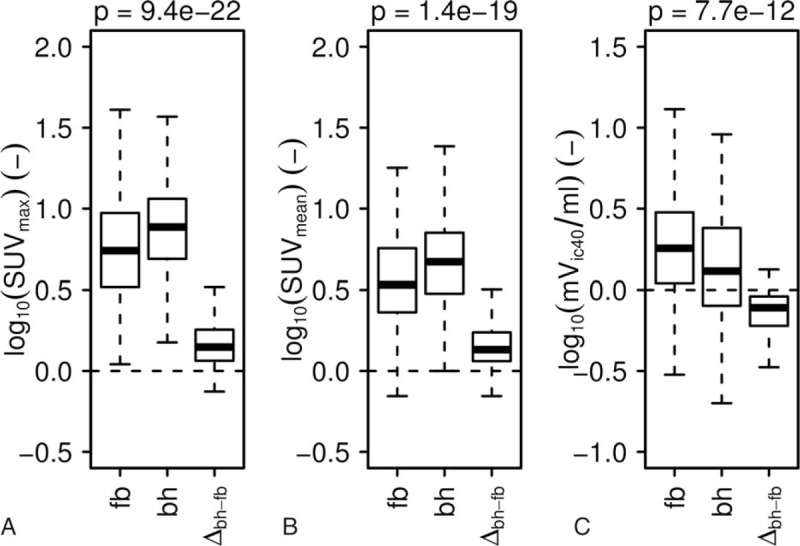
Differences in SUV_max_ (A), SUV_mean_ (B), and mV_ic40_ (C) detected by free-breathing (fb)PET and breath-hold (bh)PET. Logarithmically transformed data are illustrated by Tukey box and whisker blots, whereby boxes were used to show first, second, and third quartile, while the whiskers represent the lowest/highest value still within 1.5 interquartile ranges of the lower/upper quartile. For each parameter (A–C), the left and mid box plot show distributions for bhPET and fbPET, the right box plot shows the distribution of differences between the methods. The latter illustrates a paired *t* test between the 2 methods. *P* values are reported for each parameter (A–C).

**Figure 3:**
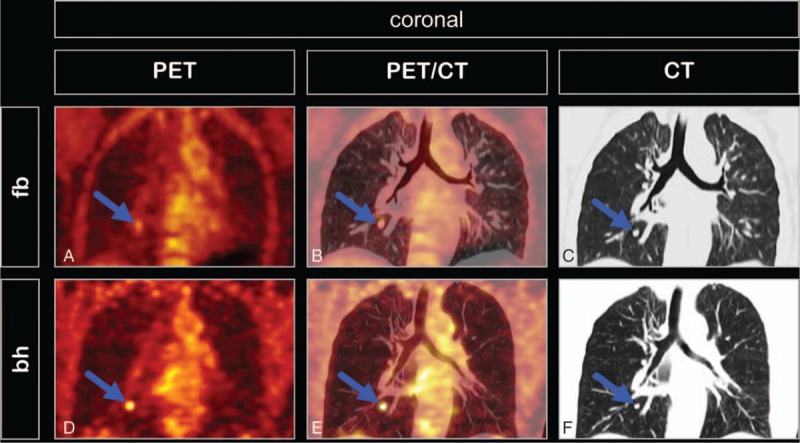
Coronal PET (A and D), PET/CT (B and E), and CT (C and F) images of a female patient with pulmonary metastasis of malignant melanoma (arrows) close to the right lower lobe bronchus. At free-breathing PET (A) the nodule appears craniocaudally elongated and blurred, with low uptake and large metabolic volume (SUV_max_ 2.3, SUV_mean_ 2.0, mV_ic40_ 3.9 mL), whereas at breath-hold PET (D) the nodule appears rounder, more sharply delineated, with higher uptake and lower metabolic volume (SUV_max_ 4.9, SUV_mean_ 3.5, mV_ic40_ 0.6 mL). Breath-hold time was 67 seconds.

The mean breath-hold time of all investigations was 43.0 ± 17.3 seconds (range 10–85 seconds). The tumors imaged with bhPET showed a significantly higher SUV_max_ compared to fbPET (7.7 ± 17.7 vs 5.5 ± 8.4; *P* < 0.001). The same was true for the SUV_mean_ (4.7 ± 11.7 vs 3.4 ± 5.3; *P* < 0.001). In contrast, the mV_IC40_ was significantly smaller at bhPET than at fbPET (1.3 ± 11.4 mL vs 1.8 ± 16.0 mL; *P* < 0.001). In terms of %bh-index, the SUV_max_ was increased by 40.4 ± 81.6% and the SUV_mean_ by 35.4 ± 90.7%. The metabolic volume was reduced by 22.2 ± 38.8%. The impact of bhPET on the SUV_max_ was larger than the impact on the SUV_mean_ (Figs. 2 and 3, Table 3).

**Table 3:**
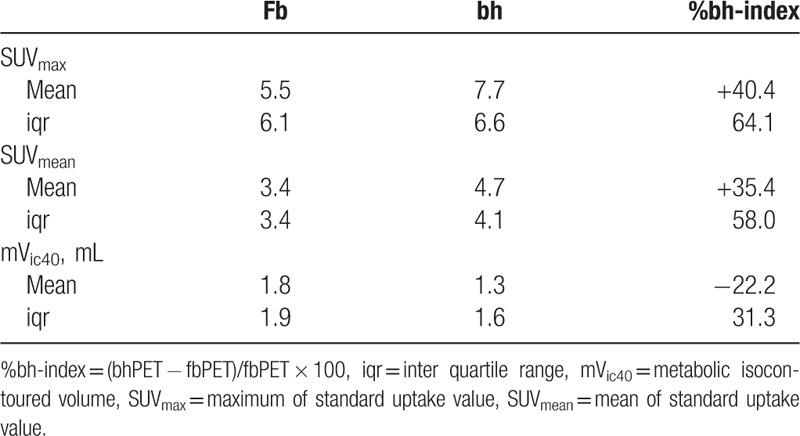
Impact of free-breathing (fb)PET and breath-hold (bh)PET on quantitative parameters (without lesion detected by bhPET only).

### 3.4 Impact of lesion size on quantitative parameters

**Figure 4:**
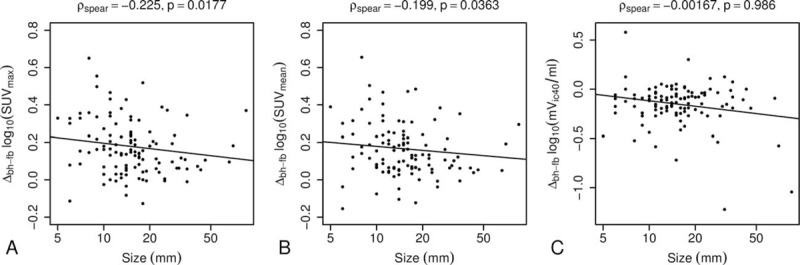
Differences of SUV_max_ (A), SUV_mean_ (B), and mV_ic40_ (C) between breath-hold PET and free-breathing PET plotted against the size of the lesions. Data were logarithmically transformed. Solid lines show the results of linear regressions. To avoid false correlations due to outliers, the Spearman rho and respective *P* values are reported for each model.

The largest lesion diameter was in mean 15.0 ± 12.6 mm (range 5–85 mm). In smaller lesions, the bhPET had significantly larger effects on SUV_max_ (ρ_spear_ =  − 0.23, *P* = 0.02) and SUV_mean_ (ρ_spear_ =  − 0.20, *P* = 0.04) than in larger lesions. The size of the lesions did not significantly affect the mV_ic40_ (ρ_spear_ =  − 0.002, *P* = 0.99) (Fig. 4).

### 3.5 Influence of time interval between fbPET and bhPET on quantitative parameters

**Figure 5:**
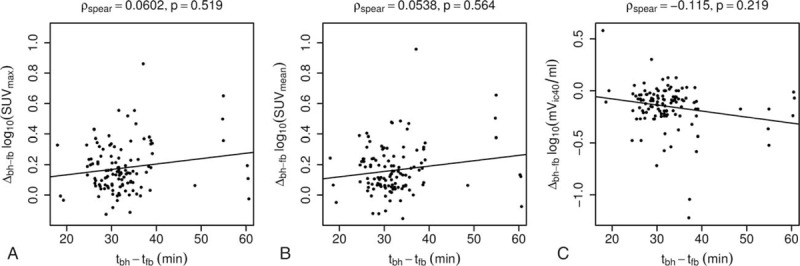
Differences of SUV_max_ (A), SUV_mean_ (B), and mV_ic40_ (C) between breath-hold PET and free-breathing PET plotted against the time interval between the scans. Data were logarithmically transformed. Solid lines show the results of linear regressions. To avoid false correlation due to outliers, the Spearman rho and respective *P* values are reported for each model.

Mean interval between fbPET and bhPET was 31 ± 7 minutes (range 17–60 minutes). The time interval had no significant influence on SUV_max_ (ρ_spear_ = 0.06, *P* = 0.52), SUV_mean_ (ρ_spear_ = 0.05, *P* = 0.56), or mV_ic40_ (ρ_spear_ =  − 0.12, *P* = 0.22) (Fig. 5).

## 4 Discussion

Breath-hold and free-breathing methods are available to limit the influence of respiratory motion during PET examinations. A bhPET would be preferable in terms of reduced time efforts and required equipment, but its feasibility largely depends on compliance and general health status of the patient.^[7]^ In this study, the bhPET technique was evaluated exclusively in patients with histologically proved malignant melanoma. The examinations were performed for staging, restaging, and surveillance, therefore only metastatic tumors were taken into account.^[1]^

Previous studies have not elaborated on the detection of additional lesions in bhPET, reports being limited to lymphangitis carcinomatosa^[8]^ and colorectal carcinoma.^[11]^

Side-by-side interpretation of fbPET and bhPET studies was preferred instead of a blinded approach. The intention of bhPET in our setting was not to replace fbPET but to use it as a supplementary option to improve general diagnostic performance. Moreover, the side-by-side appraisal corresponds to the current use in clinical routine.

In this study bhPET enabled identification of one additional liver lesion by targeted comparison with ceCT, progressive in size on follow-up imaging and therefore considered metastatic. In this patient, a histological examination was not clinical appropriate due to the presence of further multiple liver metastases. In other organs, particularly the lung, the bhPET did not identify any additional lesions. This is remarkable because, in advanced melanoma, lung metastases are actually more numerous than liver metastases, for example at stage IV only 4% of the metastases are found in the liver against 19% in the lung.^[13]^ In our patients 11% of the metastases were in the liver and 26% in the lungs (Table 2). A conceivable explanation is that additional lesions in the liver usually remain masked due to the relatively high metabolic activity of the liver parenchyma, and detection is impaired due to the blurring induced by respiration.

In principle, bhPET allows the identification of additional lesions in malignant melanoma, but the potential clinical relevance remains questionable. The scarcity of published data on this issue indicates that the relevance is also limited for other tumor entities.

In fbPET the respiratory motion in the 2-minute scanning time per bed position induces a blurring of focal lesions, which in turns leads to changes of SUV_max_, SUV_mean_, and metabolic volume. Several bhPET studies have focused on lung carcinoma, but only a few have addressed abdominal diseases, and—to our knowledge—none has explicitly reported on melanoma lesions. Also, all available studies describe SUV_max_ as quantitative marker,^[10,14,15]^ but only a few mention the metabolic volume^[10,16–18]^ or the SUV_mean_.^[10]^ In chest lesions, for example, the SUV_max_ at bhPET has proven 32.5% higher than at fbPET.^[14]^ Similar results have been obtained for abdominal lesions.^[17,18]^ Our study confirmed these data in that melanoma lesions (whole trunk) displayed a 40.4% higher SUV_max_.

The SUV_max_ is certainly a simple and robust marker of the area with the highest uptake of tracer, but sometimes it corresponds only to 1 voxel. Instead, SUV_mean_ reflects the tracer accumulation during the whole process, hence more closely reflecting the investigator's visual impression. The influence of bhPET on SUV_mean_ was investigated in only 1 study, also revealing a significantly higher SUV_mean_ compared to fbPET.^[10]^ Data on the metabolic volume are also scarce, for example at F-18-FDG bhPET this proved to be 20% smaller,^[17,18]^ given that the spatial fixation during the breath-hold phase reduces the blurring and better delineates the lesion. The relative difference found in the present study (−22.2%) is therefore well compatible with the published data. Thus, the presented data on the use of bhPET in melanoma confirm the results obtained with other tumors, that is, the more commonly used fbPET underestimates the SUV_max_ and SUV_mean_ and overestimates the mV_ic40_.

In this melanoma patient population, the size of the lesions had a significant influence on SUV_max_ and SUV_mean_ at bhPET, that is, in particular smaller lesions were more sharply delineated and easier to identify. This result confirms similar observations in lung carcinoma, that SUV_max_ differences between standard and bhPET are significantly more pronounced in small lesions.^[9,19]^ There was no statistical significance of the lesion size regarding to mV_IC40_. The lack of impact on the mV_ic40_ remains to be verified in other studies, as no comparable data are available in the literature.

A potential confounder in this study is the time interval between fbPET and bhPET (mean 31 minutes). As radiotracers have specific kinetics, the quantitative parameters can change over time. This confounder is mentioned in other F-18-FDG studies, but statistical analyses of the data have not been published.^[10,20]^

In general, the SUV is higher in malignant and granulomatous diseases compared to benign diseases, whereas over time a rapid uptake is typically followed by a plateau.^[21]^ Investigations on when the plateau is achieved in a given tumor have provided different results. An in vitro F-18-FDG study with different subtypes of cell lines of malignant melanoma has shown that the SUV_max_ steeply increases within the first 60 minutes, and then progressively less until 120 minutes postradiotracer administration.^[22]^ A clinical study on breast cancer has shown a rapid increase of uptake until 90 minutes post-F-18-FDG injection, followed by a less rapid uptake until 180 minutes.^[23]^ A dynamic study with nonsmall-cell lung carcinoma (NSCLC) has shown that a plateau was not reached before 2.5 hours after F-18-FDG administration.^[24]^ The data of the present fbPET/bhPET study were acquired at a mean of 101 and 132 minutes, respectively, with a mean time difference of 31 minutes. This implies that the investigations took place in a phase of slower tracer accumulation. Although a certain influence of the FDG kinetic cannot be excluded, the results showed that the time interval between fbPET and bhPET did not significantly influence SUV and mV_ic40_. This result is analogous to a study with PET acquisitions at 50 and 90 minutes (time difference of 40 minutes), showing that the later acquisitions had a significantly higher SUV_max_, but the time difference between the acquisitions did not play a significant role.^[25]^

The present study has some limitations. Routine PET scans are usually acquired with 2 to 3 minutes per bed position, but patients cannot hold their breath for the entire time, especially in view of their impaired health status. The shorter scan time for bhPET (mean 43 seconds, range 10–85 seconds) leads to reduced signal statistics and higher background-noise-ratio. Some authors have attempted to estimate the optimal scanning time for bhPET, given that PET scanners of different companies vary regarding technical specifications. A phantom study with PET acquisitions stopping during simulated respiratory pauses, but with addition of short breath-hold phases, showed that acquisition times of 45, 60, and 120 seconds had a significantly higher diagnostic precision than a fbPET at 120 seconds, suggesting that a breath-hold of at least 45 seconds is necessary.^[26]^ Another phantom study postulated that the breath-hold should be greater than 90 seconds, but consisting of 8 intervals of 12 seconds each.^[27]^ Since breath-hold periods with the same respiratory depth are difficult to achieve without additional intervention, other authors opted for a single episode of sufficient breath-hold, lasting for 30 seconds for investigation of large tumors,^[5]^ or 20 seconds in a phantom study.^[9]^

The present study was based on a single bhPET. Therefore, it must be considered that the respiratory depth during the PET and the CT acquisition may have differed, leading to a mismatch between the 2 examinations and also to a suboptimal attenuation correction.^[28–30]^

A further drawback of this breath-hold study was that the scanning area was limited (1 bed position, or 21.8 cm craniocaudally). The lungs, and possibly also enlarged livers, were not completely included. In addition, the upper and lower edges of the scanning area showed some imaging artifacts.

Finally, the retrospective nature of the study implied that not all patients had ceCT or ceMRI available for morphological comparison. Some of the patients, in addition, did not undergo ceCT or ceMRI because of contraindications to the use of contrast media, and only 60% had follow-up investigations for further verification.

## 5 Conclusions

In summary, the use of bhPET with F-18-FDG yielded higher SUV and lower mV_ic40_ than fbPET. On bhPET, SUV significantly depended on lesion size, but not on the time difference between fbPET and bhPET acquisitions.

The better definition obtained with the bhPET technique may become a useful diagnostic option when fine quantitative evaluations for staging, recurrence, prognosis, and therapeutic response are needed, particularly for small lesions. bhPET enabled the identification of 1 additional liver metastasis, which was not clinical relevant for the further management of this particular patient with multiple metastases, but could have been decisive for a patient without known metastasis. In this respect, bhPET may be recommendable in selected cases. Future developments of more sensitive technologies with reduced acquisition time, or use of larger detectors with larger field-of-view, may enable a wider use of bhPET as a feasible alternative to the complex rgPET method.
